# Association Between Alzheimer's Disease and Glaucoma: A Study Based on Heidelberg Retinal Tomography and Frequency Doubling Technology Perimetry

**DOI:** 10.3389/fnins.2015.00479

**Published:** 2015-12-18

**Authors:** Massimo Cesareo, Alessio Martucci, Elena Ciuffoletti, Raffaele Mancino, Angelica Cerulli, Roberto P. Sorge, Alessandro Martorana, Giuseppe Sancesario, Carlo Nucci

**Affiliations:** ^1^Ophthalmology Unit, Department of Experimental Medicine and Surgery, University of Rome Tor VergataRome, Italy; ^2^Laboratory of Biometry, Department of Systems Medicine, University of Rome Tor VergataRome, Italy; ^3^Neurology Unit, Department of Systems Medicine, University of Rome Tor VergataRome, Italy

**Keywords:** Alzheimer disease, glaucoma, optic nerve head, HRT-3, FDT, RNFL, CCT

## Abstract

**Aim:** To assess the frequency of glaucoma-like alterations in Alzheimer's disease (AD) patients using Heidelberg Retinal Tomograph III (HRT-3) and Frequency Doubling Technology (FDT) perimetry.

**Methods:** The study included 51 eyes of 51 AD subjects and 67 eyes of 67 age- and sex-matched controls. Subjects underwent an ophthalmological examination including measurements of intraocular pressure (IOP), Matrix FDT visual field testing, optic nerve head morphology and retinal nerve fiber layer thickness (RNFLt) assessment by slit-lamp biomicroscopy and HRT-3.

**Results:** The frequency of alterations was significantly higher in the AD group (27.5 vs. 7.5%; *p* = 0.003; OR = 4.69). AD patients showed lower IOP (*p* = 0.000) despite not significantly different values of central corneal thickness (CCT) between the groups (*p* = 0.336). Of all the stereometric parameters measured by HRT-3, RNFLt was significantly lower in AD patients (*p* = 0.013). This group also had significantly worse results in terms of Moorfields Regression Analysis (*p* = 0.027). Matrix showed significantly worse Mean Deviation (MD) (*p* = 0.000) and Pattern Standard Deviation (PSD) (*p* = 0.000) values and more altered Glaucoma Hemifield Test (*p* = 0.006) in AD patients. Pearson's R correlation test showed that Mini Mental State Examination is directly correlated with MD (*R* = 0.349; *p* = 0.034) and inversely correlated with PSD (*R* = −0.357; *p* = 0.030).

**Conclusion:** Patients with AD have a higher frequency of glaucoma-like alterations, as detected by the use of HRT-3. These alterations were not associated with elevated IOP or abnormal CCT values.

## Introduction

Glaucoma, the major cause of irreversible blindness worldwide, is a progressive optic neuropathy associated with degeneration of retinal ganglion cells (RGCs) and their axons, and it is characterized by a typical optic nerve appearance and corresponding visual field loss (European Glaucoma Society, 2008). Until now, increased intraocular pressure (IOP) has been considered the major treatable risk factor for the disease. Therefore, despite the relationship between the reduction of IOP and glaucomatous damage is not yet known, the achievement of an “individual” target pressure has paramount importance. However, it has been clinically observed that a significant reduction of the IOP does not always stop the disease (Leske et al., 2003). Some patients, in fact, experience a progression of glaucoma even after a significant reduction of the IOP levels, whereas others show pathognomonic alterations despite the IOP is in the normal range.

The exact pathophysiology underlying the glaucoma is currently unknown. However, studies using magnetic resonance imaging (MRI) have shown that the disease extends well beyond the eye, affecting the entire visual pathway, thus suggesting a possible connection with other neurodegenerative diseases (Nucci et al., 2013).

Interestingly, clinicians and researchers have observed close links between glaucoma and Alzheimer's Disease (AD) (Wostyn et al., 2009) whose importance is increasing as life-expectancy of populations rises.

AD is the leading cause of dementia worldwide and is estimated to affect approximately 50–60% of dementia patients. The disease is characterized by a gradual, progressive and irreversible decline in cognitive function and is associated with certain risk factors, such as genetics and vascular alterations. The presence of extracellular amyloid-β (Aβ) senile plaques and the intracellular deposition of abnormally phosphorylated tau protein are the main hallmarks of the disease (Blennow et al., 2006).

Although several studies have suggested that there is no association between glaucoma and an increased risk of developing AD (Kessing et al., 2007; Bach-Holm et al., 2012; Ou et al., 2012; Keenan et al., 2014) other population-based studies have reported a higher prevalence of glaucoma in patients affected by the disease (Chandra et al., 1986; Bayer and Ferrari, 2002; Bayer et al., 2002a,b; Tamura et al., 2006; Lin et al., 2014; Pelletier et al., 2014). These findings have been supported by data showing that inheritance of the AD-associated [epsilon]4 allele is twice as high among glaucoma patients, irrespective of the presence of ocular hypertension (Wostyn et al., 2009).

These studies may support the hypothesis that, in some patients, glaucoma could be the expression of a neurodegenerative process of the central nervous system that may be only partially influenced by ocular factors, such as the IOP (Nucci et al., 2013, 2015).

The aim of this study was to evaluate the frequency of glaucoma-like alterations in a group of patients with AD using diagnostic criteria based on Frequency Doubling Technology (FDT) perimetry and Heidelberg Retinal Tomography-3 (HRT-3).

## Materials and methods

This study adhered to the Declaration of Helsinki, and the Institutional Review Boards and Ethics Committees of Tor Vergata University Hospital approved its protocol.

Written informed consent was obtained from all participants.

The study included 51 consecutive newly diagnosed Alzheimer's disease cases (51 eyes), recruited from the Department of Neurology of Tor Vergata University Hospital.

AD diagnosis was made according to the NINCDS-ADRDA guidelines and the Diagnostic and the Statistical Manual of Mental Disorders (DMS IV) (McKhann et al., 1984; American Psychiatric Association, 2004). All patients underwent a complete clinical investigation, including medical history, neurological examination, mini mental state examination (MMSE), a complete blood screening (including routine exams, thyroid hormones, level of B12), neuropsychological examination (Pierantozzi et al., 2004) a complete neuropsychiatric evaluation and neuroimaging consisting of 1,5 T magnetic resonance imaging. All the patients were studied for ApoE genotype. Exclusion criteria were the following: (1) patients with isolated deficits and /or unmodified MMSE (≥25/30) on revisit (6, 12, 18 months follow-up), patients with clinically manifest acute stroke in the last 6 months showing an Hachinsky scale >4, and a radiological evidence of sub-cortical lesions. None of patients revealed pyramidal and/or extrapyramidal signs at the neurological examination.

The control group consisted of 67 healthy subjects (67 eyes), who were randomly recruited from the General Outpatient Clinic of the Ophthalmological Department of Tor Vergata University Hospital.

All the enrolled subjects underwent a comprehensive eye examination, including the determination of best corrected visual acuity (BCVA) with logarithmic visual acuity charts “ETDRS” (Precision Vision, la Salle USA), IOP measurement using Goldmann applanation tonometry, central corneal thickness (CCT) measurement using an ultrasound pachymeter (Pachette DGH 500; DGH Technology, Inc., Philadelphia, PA), gonioscopy, and slit-lamp biomicroscopy of the anterior and posterior segments.

Subjects with spherical refraction beyond ±5.0 D and/or cylinder correction beyond ±3.0 D, or any ocular or systemic disease, which could affect the optic nerve or the visual field examination results, were excluded from the study.

**Figure 1:**
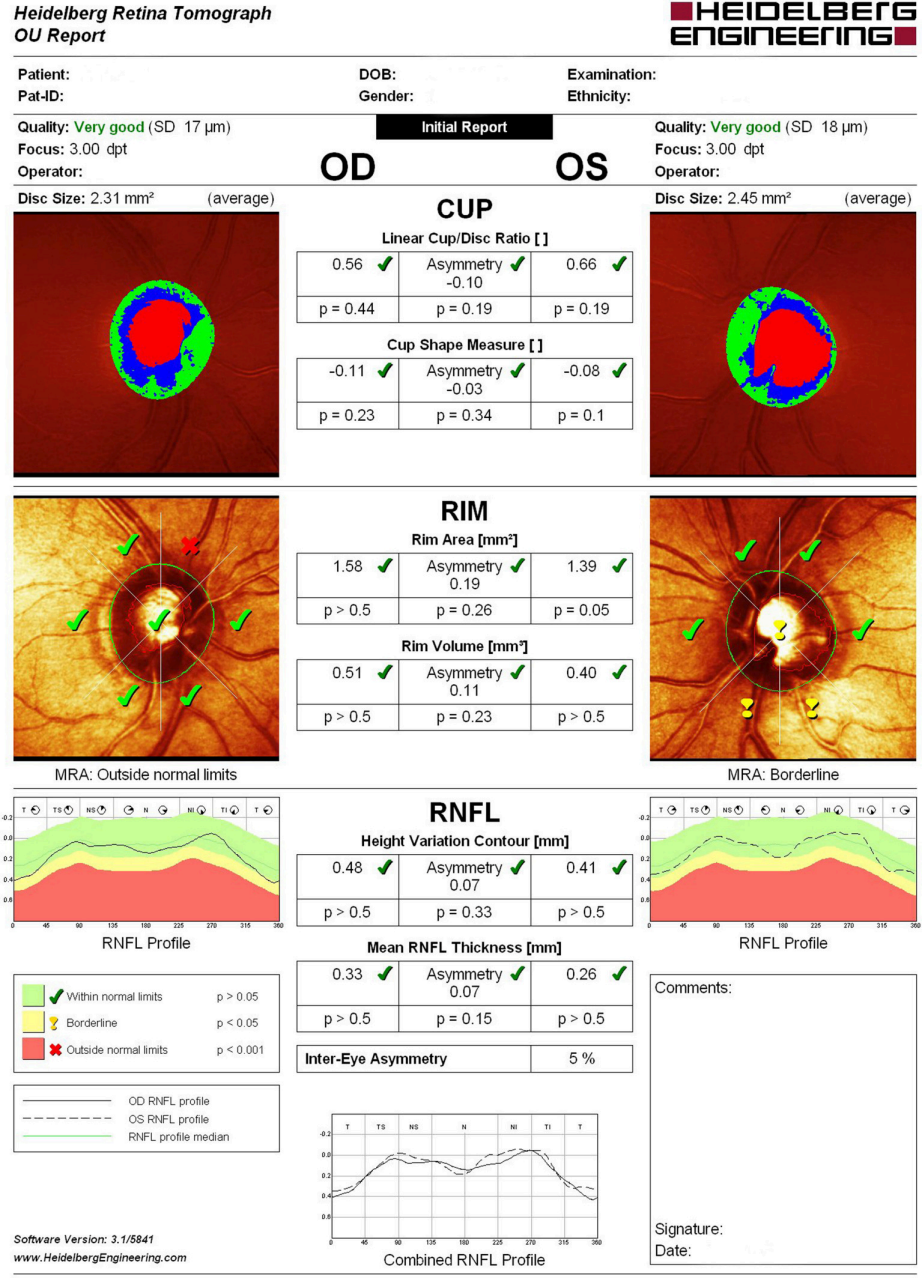
**Heidelberg retinal tomograph III (HRT-3) analisys report**.

Structural evaluation of the optic disc and retinal nerve fiber layer (RNFL) were performed using HRT-3 (Heidelberg Engineering, Heidelberg, Germany), which is a proven tool for detecting and managing glaucoma, assisting in the identification of pre-perimetric disease and monitoring of progression. In all astigmatic eyes beyond ±1.0 D, corrective cylindrical lenses were used. All participants whose tests revealed a Mean Pixel Height Standard Deviation >30 micron were excluded from the study. Disc measures of the enrolled patients did not exceed the database disc area cut-offs provided by the HRT-3 manufacturer. (Dascalu et al., 2010) The same operator using only four points manually traced a contour line around the optic disc edge (the inner edge of Elschnig's ring). For each test Moorfields Regression Analysis (MRA), Glaucoma Probability Score (GPS) and the following stereometric parameters were evaluated: retinal nerve fiber layer thickness (RNFLt), Rim Area, Rim Volume, Cup-Shape Measure (CSM), Height Variation Contour (HVC), Cup/Disc Ratio (CDR) asymmetry, and vertical Cup/Disc Ratio (vCDR) (Figure 1).

MRA results, which differentiates abnormal from healthy optic nerve heads by detecting diffuse and focal changes of the neuroretinal rim area, were defined as follows: 1 = within normal limits; 2 = borderline; 3 = outside normal limits.

GPS, which automatically identifies patterns of structural change consistent with glaucoma providing a probability of abnormality, was defined as follows: 1 = within normal limits; 2 = borderline; 3 = outside normal limits; 0 = not classified.

The optic disc appearance was also assessed by slit lamp biomicroscopy examination and defined as pathological when at least one of the following features was present: vCDR asymmetry between the eyes ≥0.2, CDR ≥0.5, neuroretinal rim thinning, splinter-shaped disc hemorrhages, notching, localized pallor, focal, or generalized peripapillary atrophy or nerve fiber layer defects, baring of circumlinear or cilioretinal vessels (European Glaucoma Society, 2008).

Matrix FDT perimetry (Welch Allyn, Skaneateles Falls, NY, USA and Carl Zeiss Meditec, Dublin, CA, USA) was obtained from all participants using the 30-2 program. Before proceeding, all patients received a training and underwent a pre-test lasting 60 s. The examination was performed on each eye. Only subjects who performed reliable visual fields (≤ 33% fixation losses, false positives, and false negatives) were included. For each test the following index were evaluated: mean deviation (MD), pattern standard deviation (PSD), and glaucoma hemifield test (GHT). MD is a measure of the average deviation from the patient's light sensitivity and that of age-matched controls. PSD shows localized loss of light sensitivity, which is one of the hallmark of glaucoma. GHT warns the clinician about the occurrence of significant differences in terms of clusters of altered points between the superior and inferior hemifields (Scuderi et al., 2008). GHT results were defined as follows: 1 = within normal limits; 2 = limit; 3 = borderline; 4 = outside normal limits; 5 = general loss of sensitivity. The severity of visual field damage was classified according to the FDT Glaucoma Staging System 2 (GSS2) (Brusini, 2006).

The same operator throughout the whole study performed all the examinations. For each patient, only the eye with the worse results in terms of RNFLt, assessed by HRT-3, was included in the study. Glaucoma-like alterations were defined as the occurrence of visual field specific defects (GSS2 stage ≥1) and morphological optic disc alterations at biomicroscopy and/or pathological changes of HRT-3 parameters.

All data were initially entered into an EXCEL database (Microsoft, Redmond, Washington—United States) and the analysis was performed using the Statistical Package for the Social Sciences, Windows version 19.0 (SPSS, Chicago, Illinois, USA).

Gaussian distributions were differentiated from non-Gaussian ones by the use of the Kolmogorov-Smirnov test. Descriptive statistics consisted of the mean ± standard deviation (SD) for parameters with Gaussian distribution or frequencies (%) for occurrences. Gaussian parameters were analysed using one-way ANOVA test. Conversely, non-Gaussian parameters were analysed using Mann Whitney U or Kolmogorov-Smirnov Z tests. For categorical variables, comparison of frequencies among groups was performed using the Chi-Square test or Fisher's exact test. Correlations among ocular parameters (MD, PSD, GHT, MRA, RNFL, IOP) and MMSE were performed using Pearson's R correlation test. A *p*-value < 0.05 was considered statistically significant.

## Results

Descriptive analysis of the AD and Control Groups is shown in Table 1.

**Table 1:** **Descriptive analysis of the Alzheimer and control groups**.

**Group**	**Sex**	**Eyes**	**Mean age ± SD**	**Median**	**Min**	**Max**	**MMSE**
CTRL	M	29	69.9 ± 5.9	70	58	80	–
	F	38	68.2 ± 5.9	68	58	80	
	Total	67	68.9 ± 5.8	69	58	80	
AD	M	25	69.7 ± 6.6	69	58	80	20.8 ± 4.8
	F	26	71.4 ± 5.9	72	58	80	20.8 ± 3.6
	Total	51	70.6 ± 6.2	71	58	80	
P		0.536^a^	0.152^a^				

*AD, Alzheimer Group; CTRL, Control Group; MMSE, Mini Mental State Examination; SD, standard deviation; p < 0.05*.

a *ANOVA One-way test*.

All of the patients resulted E3/E4 in ApoE genotype study. No significant differences between the groups were found when considering sex (ANOVA One-way test; *p* = 0.536) and age (ANOVA One-way test; *p* = 0.152).

The frequency distribution of eyes with Matrix visual field alterations compatible with glaucoma associated with optic disc damage and/or HRT-3 assessed alterations was significantly higher in the AD group than in controls (27.5 vs. 7.5%; Chi-Square test; *p* = 0.003; OR = 4.69).

Remarkably, the study revealed that the mean IOP values of the two groups were in the normal range and, more interestingly, that AD patients had even lower IOP values than controls (ANOVA one-way test; *p* = 0.000). Moreover, the analysis of mean CCT values revealed no statistically significant difference between the groups (ANOVA One-way test; *p* = 0.336) (Table 2) ensuring an accurate measurement of the IOP.

**Table 2:** **Statistical analysis of the ophthalmological parameters considered**.

**Variable**	**AD**	**CTRL**	***P***
IOP	13.31 ± 2.00	14.81 ± 2.25	**0.000^a^**
CCT	538.94 ± 35.34	544.84 ± 30.83	0.336^a^
RNFL	0.17 ± 0.06	0.20 ± 0.58	**0.013^a^**
Rim volume	0.29 ± 0.12	0.33 ± 0.13	0.063^a^
Rim area	1.35 ± 0.30	1.42 ± 0.35	0.271^a^
CSM	−0.17±0.13	−0.18±0.06	0.447^a^
HVC	0.30 ± 0.08	0.33 ± 0.07	0.141^a^
vCDR	0.46 ± 0.19	0.43 ± 0.17	0.416^a^
CDRa	0.07 (0.0; 0.13)	0.05 (0.0; 0.32)	0.339^b^
MRA	1.6 (1.0; 3.0)	1.3 (1.0; 3.0)	**0.027^b^**
GPS	1.0 (1.0; 3.0)	1.0 (1.0; 3.0)	0.208^b^
MD	−6.19±0.84	−2.82±0.49	**0.000^a^**
PSD	4.66 (1.91; 10.63)	3.03 (0.18; 5.28)	**0.000^b^**
GHT	4.0 (1.0: 4.0)	2.0 (1.0; 4.0)	**0.006^b^**

*Descriptives: Mean ± SD for gaussian variables and Median (min.; max.) for non-gaussian variables. IOP, Intraocular Pressure (mmHg); CCT, Central Corneal Thickness (μm); RNFL, Retinal Nerve Fiber Layer; CSM, Cup Shape Measure; HVC, Height Variation Contour; vCDR, Vertical cup/disc ratio; MRA, Moorfields Regression Analysis; GPS, Glaucoma Probability Score; MD, Mean Deviation; PSD, Pattern Standard Deviation; GHT, Glaucoma Hemifield Test; CDRa, Cup/Disc Ratio asymmetry; AD, Alzheimer Group; CTRL, Control Group; p < 0.05*.

a *ANOVA One-way test*.

b *Mann-Whitney U test*.

Comparison of HRT-3 stereometric parameters between the groups showed significantly reduced RNFLt values (ANOVA One-way test; *p* = 0.013) in the AD group (Table 2). Contrastingly, no significant differences were found when the following stereometric parameters were considered: Rim area (ANOVA One-way test; *p* = 0.271); CSM (ANOVA One-way test; *p* = 0.447); HVC (ANOVA One-way test; *p* = 0.141); Cup/Disc Ratio asymmetry (CDRa) (Mann Whitney U test; *p* = 0.399) and vCDR (ANOVA One-way test; *p* = 0.416). An almost significantly difference was found when RIM Volume was considered (ANOVA one-way test; *p* = 0.063). In addition, the statistical analysis, as shown in Table 2, reveals a significantly worse MRA classification in the AD group (Mann Whitney U test; *p* = 0.027), but no significant differences in terms of GPS score (Mann Whitney U test; *p* = 0.208).

When the two global FDT perimetry indices were analysed, the mean MD values (ANOVA one-way test; *p* = 0.000) and mean PSD values (Mann Whitney U test; *p* = 0.000) resulted significantly higher in the AD group (Table 2). Finally, the GHT score was significantly higher in patients with AD than in controls (Mann Whitney U test; *p* = 0.006) (Table 2).

**Figure 2:**
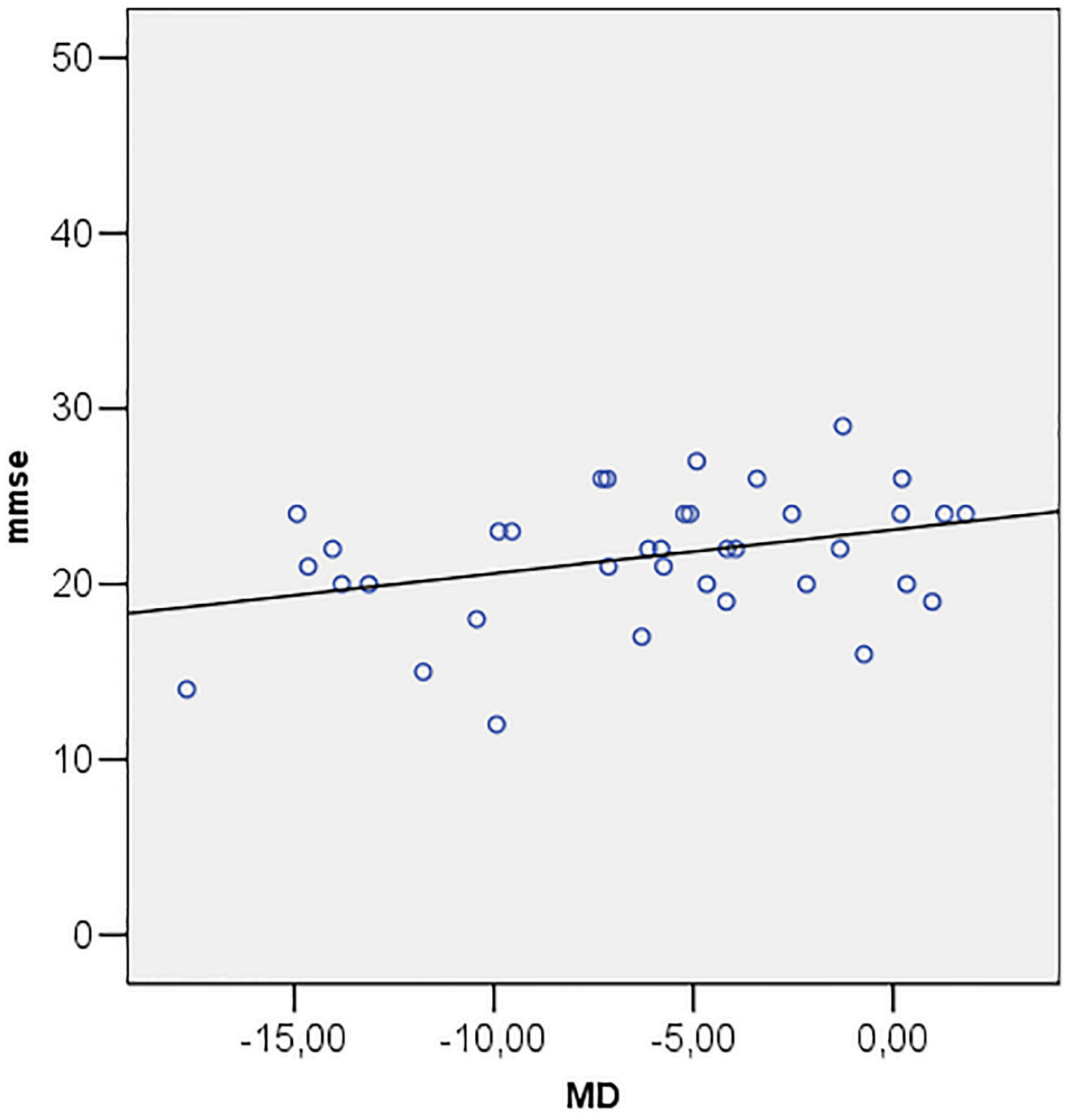
**Scatter plot of mini mental state examination (mmse) vs. mean deviation (MD)**.

**Figure 3:**
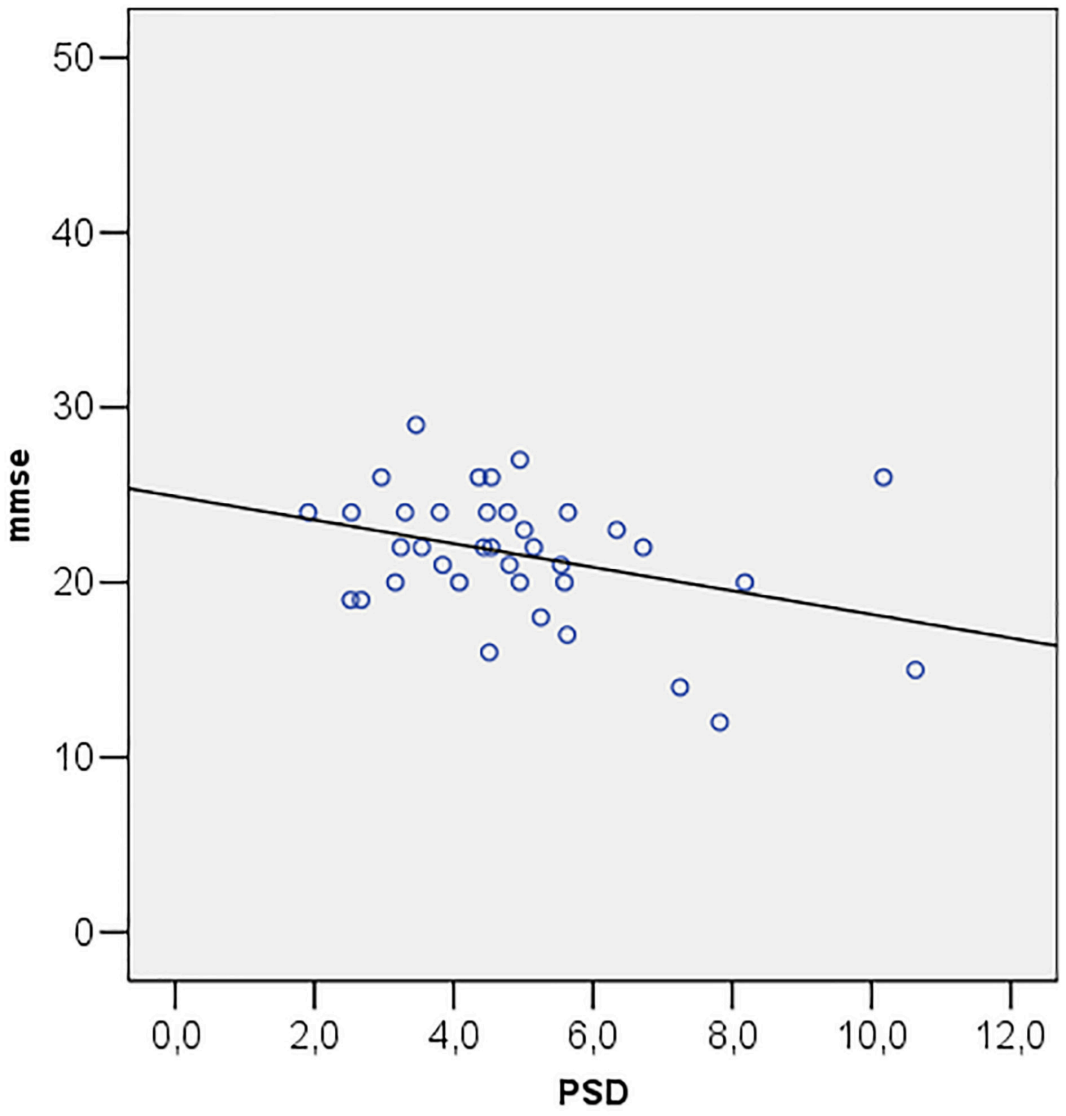
**Scatter plot of mini mental state examination (mmse) vs. pattern standard deviation (PSD)**.

Correlations among MMSE and ocular parameters (MD, PSD, GHT, MRA, RNFL, IOP) were performed using Pearson's R correlation test. The test showed that Mini Mental State Examination is directly correlated with MD (*R* = 0.349; *p* = 0.034) (Figure 2) and inversely correlated with PSD (*R* = −0.357; *p* = 0.030) (Figure 3).

## Discussion

Recent clinical studies have indicated a possible epidemiological link between glaucoma and AD, suggesting that patients suffering from the latter have an increased risk of developing glaucoma and tend to manifest the disease with a more severe clinical picture (Bayer and Ferrari, 2002; Bayer et al., 2002a,b; Tamura et al., 2006; Lin et al., 2014; Pelletier et al., 2014). However, several studies have produced divergent results (Bach-Holm et al., 2012; Keenan et al., 2014). It is important to point out that several bias, such as diagnosis of POAG made by different clinicians using non-standardized diagnostic criteria, small sample size with wide confidence limits, affected some of these studies. Unlike previous reports, in this paper diagnosis of glaucoma was based on the occurrence of standardized diagnostic criteria, such as typical visual field defects and optic nerve changes and/or altered HRT-3 parameters.

Patients with cognitive impairment are less likely to produce reliable visual field test results than healthy subjects do (Trick et al., 1995). For this reason, previous studies, attempting to use the Standard Automated Perimetry (SAP) with Humphrey Field Analyser, have found that a high percentage of patients could not perform the visual field test. As a result, in those papers diagnosis of glaucoma relied only on ophthalmoscopic and stereometric examination of the optic disc. In this study the analysis of visual field parameters has been assessed by Matrix FDT, showing typical alterations of glaucoma, such as worse results in terms of MD, PSD, and GHT, in patients with AD. In addition, MD and PSD showed, respectively, a direct and an inverse correlation with MMSE in the presence of reliable visual field tests. Methodological justification for the use of Matrix FDT is grounded on its short test duration, on its reduced “learning effect” on test outcomes, and on Matrix FDT reported ability to identify glaucomatous visual field defects earlier than SAP (Cello et al., 2000; Pierre-Filho Pde et al., 2010). Overall, these features make the test more suitable in AD patients.

Furthermore, in contrast with previous reports (Kurna et al., 2014) morphological parameters of the optic nerve head and RNFLt, assessed by a standardized technique such as HRT-3, demonstrated statistically significant differences between AD and control group. In particular, in patients with AD, RNFLt was significantly reduced, and MRA was more frequently altered compared to controls. Many studies have demonstrated the ability of HRT to detect early structural alterations of the optic nerve, achieving results comparable to those obtained by glaucoma specialists (Deleón-Ortega et al., 2006). Interestingly, according to the literature, RNFLt is one of the stereometric parameters best fitting glaucomatous damage and its progression, showing high sensitivity and specificity (Uchida et al., 1996; Trick et al., 2006). Moreover, although MRA and GPS have demonstrated adequate specificity and sensitivity, some discs cannot be classified using GPS; thus endorsing a greater usefulness of MRA in glaucoma diagnosis (Andersson et al., 2011).

In the present paper, we have observed a 27.5% frequency of glaucoma-like alterations among AD patients, a value five times higher than in controls. These data strongly support the hypothesis that a significant percentage of patients with AD have a clinical picture similar to that found in glaucoma. Interestingly, we found a statistically significant difference in the mean IOP between the groups. Besides, AD group showed IOP values even lower than controls, thus supporting the hypothesis of an increased susceptibility to the IOP of optic nerve head of patients with AD. To ensure an accurate evaluation of the IOP, in contrast to previous studies that never carried out this test, we measured the CCT values. The analysis of CCT is of paramount importance because this is an independent risk factor for developing glaucoma and, even more, is a possible major source of overestimation (or underestimation) of the IOP value assessed by applanation tonometry. The finding that AD patients had CCT values not significantly different from those of the control population, as well as from the standard, ensure the absence of any bias in IOP measurement. This is important because, in our paper, a high proportion of AD patients presented clinical features similar to those found in glaucoma, with the exception of ocular hypertension. As a result, patients with Alzheimer's disease seem to present a reduction of the RNFLt and of the rim volume regardless of the ocular hypertension. It is therefore conceivable that this reduction could be the result of the extensive central neuronal loss, typical of Alzheimer's disease, independent of the IOP, which could affect the entire visual pathways. Consequently, in some cases, the clinical picture that we currently define as glaucoma may actually be the expression of a neurodegenerative disease of the central nervous system affecting, by transynaptic degeneration, the entire visual pathway altering the performance of important vision-related functions and quality of life (Nucci et al., 2013; Martucci et al., 2014; Cesareo et al., 2015). These results have been also confirmed by a recent meta-analysis, and other studies, that reported a significant RNFLt (He et al., 2012; Marziani et al., 2013) and macular volume reduction, assessed by optical coherence tomography (OCT), in AD when compared to healthy subjects (Gao et al., 2015). These data suggest a possible usefulness of the OCT in diagnosis of neurodegenerative disease such as AD (Larrosa et al., 2014; Rebolleda et al., 2015).

The mechanisms underlying the association between AD and glaucoma are the subject of intense debate in the literature. Interestingly Wostyn et al. (2009) reported a significant rate (25%) of very low cerebrospinal fluid pressure (CSFP) values in AD patients. Incidentally, this percentage is similar to that of glaucoma-like alterations found in this paper in AD patients. For anatomical reasons, IOP is counterbalanced by CSFP and optic nerve tissue pressure from the retro-laminar regions. It has been hypothesized that a reduction in CSFP can bring about a displacement of the lamina cribrosa, resulting in axonal damage at this point. Furthermore, clinical studies on NTG patients showed a significantly lower CSFP and a higher trans-lamina cribrosa pressure in these patients compared to healthy subjects. Therefore, the CSFP reduction may play a role in the optic nerve damage observed in patients with AD (Wostyn et al., 2009). This might be a possible mechanism explaining why, despite significantly lower IOP values, patients suffering from AD considered in this study had higher prevalence of glaucoma-like alterations than controls.

A second hypothesis, which may explain the link between the two diseases, is that the decrease in production and turnover of the CSF observed in patients with AD could reduce the clearance of toxic substances in the subarachnoid space surrounding the optic nerve, thus activating neuroinflammatory processes (Killer et al., 2008; Ho et al., 2012). In this regard, it has been recently described the case of a glaucoma patient with medically controlled IOP who experienced a progression of the disease concomitantly with the onset of mild cognitive impairment. Interestingly, lumbar puncture revealed decreased Aβ, and elevated levels of total and phosphorylated tau (Nucci et al., 2011). It is therefore possible that deposits of tau and/or other toxic molecules also contributed to development and progression of glaucoma in patients with AD included in this study.

The cytotoxic effect of these substances has also been confirmed at cellular level by studies on autophagy (Hara et al., 2006; Levine and Kroemer, 2008; Jaeger and Wyss-Coray, 2010; Wong and Cuervo, 2010; Rodríguez-Muela and Boya, 2012). A reduction of Beclin-1, a gene product involved in the initiation and execution of autophagy, has been reported in AD patients. This seems to be associated with the accumulation of amyloid precursor protein and Aβ and, hence, neuronal cell death. In this regard, it has been recently observed that an acute rise of IOP, reducing Beclin-1, might derange the retinal autophagic machinery that constitutively occur in RGCs, causing their death (Russo et al., 2013). Therefore, all these mechanisms might have contributed to the morphological and functional damage detected by HRT and Matrix FDT.

## Conclusion

In conclusion, our data, collected using objective and standardized criteria, strongly support a link between AD and a higher risk of developing glaucoma-like alterations even without elevated IOP levels. Considering that epidemiological estimates are forecasting an exponential increase in Alzheimer's disease over the next 20 years, there is a risk that in the future we will face a large number of patients with optic nerve head and RNFLt alterations linked to this neurodegenerative disease.

## Author contributions

MC, AM, EC, RM, AC, AM, GS, CN: Substantial contributions to the conception or design of the work; the acquisition, analysis, and interpretation of data for the work; drafting the work and revising it critically for important intellectual content; final approval of the version to be published; agreement to be accountable for all aspects of the work in ensuring that questions related to the accuracy or integrity of any part of the work are appropriately investigated and resolved. RS: analysis and interpretation of data for the work; drafting the work and revising it critically for important intellectual content; final approval of the version to be published; agreement to be accountable for all aspects of the work in ensuring that questions related to the accuracy or integrity of any part of the work are appropriately investigated and resolved.

### Conflict of interest statement

The authors declare that the research was conducted in the absence of any commercial or financial relationships that could be construed as a potential conflict of interest.
